# Modeling antibody persistence after MenACYW-TT vaccination and comparative analysis with other quadrivalent meningococcal vaccines

**DOI:** 10.1038/s41598-025-08112-0

**Published:** 2025-07-10

**Authors:** Laurent Coudeville, Isabelle Bertrand-Gerentes, Céline Zocchetti, Edith Langevin, Siham Bchir, Florence Coste, Philipp Oster

**Affiliations:** 1 Global Medical, Sanofi Vaccines, 14 Espace. Henry Vallée, 69007 Lyon, France; 2 Global Biostatistical Sciences, Sanofi Vaccines, Marcy L’Étoile, France

**Keywords:** Invasive meningococcal disease, Quadrivalent meningococcal vaccines, Serogroups A, C, W, Y, Long-term immunopersistence, Seroprotection, Modelling, MenACYW-TT, Vaccines, Vaccines

## Abstract

Evaluating the persistence of antibody titers produced by quadrivalent meningococcal vaccines is crucial for determining the optimal timing for primary and booster doses. Using 3 − 7-year persistence data after a single priming dose of MenACYW-TT to fit a statistical model of antibody decay over time (10 years), this analysis modeled long-term antibody persistence for this vaccine in toddlers (12–23 months), adolescents/young adults (13–26 years), and older adults (≥ 56 years), comparing it with other vaccines (MCV4-TT, MenACWY-CRM, MPSV4). The statistical model is based on a Bayesian approach and it accounts for vaccine- and age group-specific antibody decline, missing data, assay errors, and antibody boosting from breakthrough infections. At 10 years post-vaccination, it predicted comparable or higher seroprotective immunopersistence for MenACYW-TT (titers ≥ 1:8 with hSBA) versus (1) MCV4-TT in toddlers (77% [95% CI 70–84] vs. 17% [6–31] for serogroup C, 67% [59–74] vs. 36% [20–53] for serogroup W); (2) MenACWY-CRM in adolescents/young adults (63% [55–71] vs. 40% [32–48] for serogroup C, 67% [59–74] vs. 57% [47–67] for serogroup W); and (3) MPSV4 in older adults (31% [23–39] vs. 22% [14–29] for serogroup C, 38% [31–46] vs. 20% [14–27] for serogroup W). In conclusion, our analysis indicated similar or higher immune persistence at 10 years for MenACYW-TT compared with other quadrivalent meningococcal vaccines, particularly for serogroups C and W.

**Supplementary Information:**

The online version contains supplementary material available at 10.1038/s41598-025-08112-0.

## Introduction

Globally, invasive meningococcal disease (IMD), caused by the bacterium *Neisseria meningitidis*, is a major cause of meningitis and septicemia^1,2^. The highest rates of IMD are seen in infants, adolescents/young adults and the elderly^3^, while higher IMD-associated case-fatality rates are seen in older adults than in other age groups^1,4^. To date, 12 serogroups of *N. meningitidis* have been identified, of which six serogroups (A, B, C, W, X, and Y) are the main cause of IMD cases worldwide^5–7^.

Quadrivalent meningococcal conjugate vaccines against serogroups A, C, W, and Y (MCV4) are widely used to prevent disease and transmission, particularly in countries where serogroups C, W, and Y are responsible for a substantial burden of disease^8^. Routine vaccination with these vaccines has successfully reduced the incidence of IMD in many countries^5,6,9–11^. Accordingly, many countries recommend routine vaccination of toddlers and/or adolescents. In the United States (US), the Advisory Committee on Immunization Practices (ACIP) recommends routine primary vaccination with a MCV4 in children aged 11 or 12 years, followed by a booster dose at age 16^12^; however, there is great variability in the schedule of routine national vaccination programs targeting infants/toddlers and adolescents among the EU and other countries worldwide, including the UK and Australia^13–15^.

Four quadrivalent meningococcal vaccines are currently licensed for different age groups in different regions. MCV4 conjugated to the diphtheria protein CRM197 (MenACWY-CRM; Menveo^®^, GlaxoSmithKline, Italy) is licensed in the USA for individuals aged between 2 months and 55 years^16^ and in Europe for individuals aged from 2 years, with no upper age limit^17^. MCV4 conjugated to diphtheria toxoid protein carrier (MCV4-DT; Menactra^®^, Sanofi Pasteur) was licensed in the USA and other countries for the prevention of IMD in individuals aged between nine months and 55 years^18^ but is no longer available in the USA and is not licensed in the EU. MCV4 conjugated to tetanus toxoid protein carrier (MCV4-TT; Nimenrix^®^; Pfizer Europe, Belgium) is licensed in Europe from age 6 weeks, with no upper age limit^19–21^, but is not licensed in the USA. For clarification and to avoid any confusion with MenACYW-TT, we refer to Nimenrix as MCV4-TT, as cited in the data source article^22^, instead of the alternative nomenclature (MenACWY-TT) that is used in some manuscripts^19–21^. Finally, MCV4 conjugated to a tetanus toxoid protein carrier (MenACYW-TT; MenQuadfi^®^, Sanofi Pasteur, USA) is licensed in the USA for individuals aged 24 months and older^23^ and in Europe for individuals aged 12 months and older^24^. Previously, a meningococcal quadrivalent polysaccharide vaccine (MSPV4; Menomune, Sanofi, USA) was licensed in the USA and globally in individuals aged 2 years and older, including older adults aged ≥56 years; however, this vaccine was discontinued worldwide in 2017.

Several published studies have reported on the immune persistence following primary vaccination with MenACYW-TT in the different age groups most affected by IMD. In a Phase 3, open-label multi-center study (NCT03476135)^22^, Piazza et al. reported the immunogenicity and safety of a booster dose of MenACYW-TT and immune persistence of the primary series vaccine in preschool children who were primed 3 years earlier at age 12–23 months with either MenACYW-TT or MCV4-TT in a Phase 2 study (NCT00427908)^25^. In a Phase 3 randomized study (NCT04084769)^26^, Zambrano et al. reported the immunogenicity and safety of a MenACYW-TT booster dose (with or without MenB vaccine) and immune persistence in adolescents and young adults aged ≥13 to <26 years who received primary vaccination with MenACYW-TT or MenACWY-CRM between 3 and 6 years previously as part of two Phase 2 studies (NCT02842853 and NCT02199691)^27,28^. In a Phase 3 randomized study^29,30^ (NCT04142242), Robertson et al. reported immune persistence in older adults (≥59 years) primed with either MenACYW-TT or with a, now discontinued, quadrivalent meningococcal polysaccharide vaccine MPSV4 (Menomune, Sanofi)^31^, either 3 years before, 5 years before, or up to 7 years before in a Phase 3 study (NCT02842866)^32^ and a Phase 2 study (NCT01732627)^33^. The immunogenicity and safety of a MenACYW-TT booster dose 5 years after primary vaccination with Men ACYW-TT at age 12–23 months were reported in the Phase 3b, open-label multicenter study (NCT04936685)^34^ following primary vaccination 5 years earlier in a Phase 3 study (NCT01732627)^35^ .

Although predefined serological correlates against meningococcal disease are widely used to define individual protection and are accepted by regulatory agencies in the setting of clinical trials^36^, it should be noted that, in real-world practice, serological correlates are not a strict binary classification (all or none) and hSBA titers <1:4 do not necessarily indicate a lack of protection^37,38^. As expected, in all these studies immunity had waned in the period between the primary vaccination and before the booster injection. However, geometric mean titers (GMTs) before the booster dose for all serogroups were higher than before the primary vaccination and the proportion of participants with serum bactericidal assay using human complement (hSBA) titers ≥1:8 was also higher than before the primary vaccination, indicating some remaining level of seroprotection against IMD^22,26,29,39^.

Using reported immune persistence data for between 3 years and 7 years after primary vaccination to fit a statistical model of antibody decay over time, the aim of this analysis was to model the long-term (up to 10 years) antibody persistence following a primary vaccination with a single dose of MenACYW-TT in the different age groups, and to compare this with the immune persistence of other quadrivalent meningococcal vaccines used in these trials.

## Methods

### Analysis data

#### Participants

Persistence data were obtained from four Phase 3 clinical reference studies^22,26,29,39^ (Table 1) in a similar manner to that used in the structured benefit–risk assessment of MenACYW-TT in individuals aged 12 months and older^36^. The reference studies assessed antibody persistence for each serogroup following primary vaccination with MenACYW-TT or another quadrivalent meningococcal (conjugated or not) vaccine at least 3 years before. The first vaccine time point (T1) was recorded at 30 days after the primary vaccination; the first long-term persistence data point (T2) was recorded between 3 and 7 years after the primary vaccination; an additional long-term persistence data point (T3) was also recorded at 5 years after primary vaccination in a subset of older adults who received MPSV4 or MenACYW-TT (Table 2). Data were analyzed according to age categories at the time of primary vaccination in the reference studies (toddlers aged 12–23 months, adolescents/young adults aged ≥ 13 to < 26 years, and older adults aged ≥  56 years).

**Table 1:** Studies reporting on the persistence of immunogenicity following primary vaccination with a single dose of a MCV4 vaccine.

Study assessing the primary dose	Country	Age at primary dose	Vaccines used as primary dose	Study assessing antibody persistence	Study design	Time between primary dose and antibody persistence assessment
Vesikari et al. (NCT00427908)^25^ March 2015 to August 2015	Finland	Toddlers: 12–23 months	MenACYW-TT (MenQuadfi®) MCV4-TT (Nimenrix^®^)	Piazza et al. (NCT03476135)^22^ February 2018 to September 2018	Phase 3, randomized, modified double-blind, immune-persistence and booster study in children	3 years
van der Vliet et al. (NCT02955797)^35^ February 2017 to October 2017	Finland, Germany, Hungary, and Spain	Toddlers: 12–23 months	MenACYW-TT (MenQuadfi®)	Martinón-Torres et al. (NCT04936685)^34^ August 2022 to February 2023	Phase 3b, open-label, multi-center study in children	5 years
Dhingra et al. (NCT02842853)^28^ July 2016 to February 2017 Chang et al. (NCT02199691)^27^ July 2014 to October 2015	USA and Puerto Rico	Naïve adolescents/young adults : ≥ 13 to < 26 years	MenACYW-TT (MenQuadfi®) MenACWY-CRM (Menveo^®^)	Zambrano et al.^26^ (NCT04084769) September 2019 to September 2020	Phase 3b randomized, modified double-blind, immune-persistence and booster study in adolescents and adults	3–6 years
Esteves-Jarmillo et al. (NCT02842866)^32^ July 2016 to February 2017 Kirstien et al. (NCT01732627)^33^ November 2012 to January 2013	USA and Puerto Rico	Naïve older adults: ≥ 56 years	MenACYW-TT (MenQuadfi®) MPSV4 (Menomune^®^) MenACYW-TT (MenQuadfi®) MPSV4 (Menomune^®^)	Robertson et al. (NCT04142242)^29,30^ October 2019 to April 2020	Phase 3, randomized, open-label, immune-persistence and booster study in older adults	3 years 5 years 6–7 years

**Table 2:** Summary of number of participants at primary vaccination included in the analysis by treatment arm and reference study.

Age group	Arm	Study assessing the primary dose	Study assessing the persistence data	N	T1 n (%)	T2 n (%)	T3 n (%)
Adolescents/young adults	MenACYW-TT	Dhingra et al. (NCT02842853)^28^	Zambrano et al. (NCT04084769)^26^	139	137 (98.6%)	137 (98.6%)	N/A
Adolescents/young adults	MenACYW-TT	Chang et al. (NCT02199691)^27^		241	239 (99.2%)	239 (99.2%)	N/A
Adolescents/young adults	MenACWY-CRM	Dhingra et al. (NCT02842853)^28^		56	3 (5.6%)	3 (5.6%)	N/A
Adolescents/young adults	MenACWY-CRM	Chang et al. (NCT02199691)^27^		134	132 (98.5%)	132 (98.5%)	N/A
Older adults	MPSV4	Kirstein et al. (NCT01732627)^33^	Robertson et al. (NCT04142242)^29,30^	26	26 (100.0%)	26 (100.0%)	N/A
Older adults	MPSV4	Esteves-Jarmillo et al. (NCT02842866)^32^		170	168 (98.8%)	168 (98.8%)	23 (13.5%)
Older adults	MenACYW-TT	Kirstein et al. (NCT01732627)^33^		60	58 (96.7%)	58 (96.7%)	N/A
Older adults	MenACYW-TT	Esteves-Jarmillo et al. (NCT02842866)^32^		215	212 (98.6%)	212 (98.6%)	28 (13.0%)
Toddlers	MenACYW-TT	Van der Vilet et al. (NCT02955797)^35^	Martinón-Torres et al. (NCT04936685)^34^	208	208 (100.0%)	208 (100.0%)	N/A
Toddlers	MenACYW-TT	Vesikari et al. (NCT00427908)^25^	Piazza et al. (NCT03476135)^22^	42	42 (100.0%)	42 (100.0%)	N/A
Toddlers	MCV4-TT	Vesikari et al. (NCT00427908)^25^		49	49 (100.0%)	49 (100.0%)	N/A

The first vaccine time point (T1) was recorded at 30 days after primary vaccination; the first long-term persistence data point (T2) was recorded at 3 years after primary vaccination for older adults and between 3 and 7 years after primary vaccination for adolescents/young adults and toddlers; an additional long-term persistence data point (T3) was also recorded at 5 years after primary vaccination in older adults who received MPSV4 or MenACYW-TT. N/A, not available.

#### Immunogenicity assessments

The immunogenicity assessments are described in detail in the reference publications^22,26,29,39^. Briefly, antibody persistence was assessed in blood samples taken prior to administration of a booster vaccination with MenACYW-TT and paired with data obtained at T1, T2 and T3.

Titers of antibodies against meningococcal serogroups A, C, W, and Y were assessed using a serum bactericidal assay using human complement (hSBA) at a single laboratory (Sanofi Global Clinical Immunology, Sanofi, Swiftwater, PA, USA). GMTs were calculated, as described in the reference publications. Seroprotection for serogroups A, C, W and Y was defined as hSBA titer ≥1:8, which was considered a conservative serological correlate of individual protection against meningococcal disease that is also widely used and accepted by regulatory agencies^36^.

### Statistical modeling

Two statistical models (Model 1 and Model 2) based on a Bayesian approach from log-transformed titers (base 2) were implemented to fit serogroup-specific antibody decline over time and predict the trajectory of titers over 10 years. In both models, antibody decline was modelled using a log-logistic approach, which enabled us to account for an initial rapid decline in the first phase followed by a slower decline^40^. This pattern is consistent with observations of antibody persistence data for meningococcal^41,42^ and other vaccines^43,44^.

The estimation of the parameters of these models was performed for all age groups simultaneously but we considered specific antibody decline function by group, as defined by age group, reference study, and vaccine used. We also assessed possible interactions between serogroups by using a multivariate normal distribution for the decline between two time points, and considered the impact of time for the variance associated with antibody decline. Assay measurement error was included by considering the actual titer to be in the range of 1 dilution of observed titer (e=0.5). Finally, in some participants we observed an increase rather than a decrease between two consecutive time points. This led us to include in Model 2 the impact of natural exposure to the pathogen and to estimate the corresponding frequency of occurrence and impact on titers where this occurred. The two models are described below.

Model 1, which focused on antibody decline but accounts for correlation between serogroups and assay measurement error, is defined as follows:

$$\left\{ {\begin{array}{*{20}l} {[Z_{ij}^{\beta } ]\sim {\mathcal{N}}(\mu_{ij}^{\beta } ,(t_{ij} - t_{ij - 1} )\Omega^{S} )} \hfill \\ {Z_{ij}^{\beta } = X_{ij - 1}^{\beta } - X_{ij}^{\beta } } \hfill \\ {\mu_{ij}^{\beta } = X_{ij - 1}^{\beta } \left( {1 - \frac{{\alpha g + t_{ij - 1}^{\beta g} }}{{\alpha g + t_{ij}^{\beta g} }}} \right)} \hfill \\ {X_{ij}^{\beta } = \tilde{X}_{ij}^{\beta } + \in_{ij} } \hfill \\ { \in_{ij} \sim {\text{Uniform}}( - e,e)} \hfill \\ \end{array} } \right.$$

where:

$$\begin{aligned} & X_{{ij}}^{s} :{\text{Actual log - transformed titer for subject }}i{\text{ at data point }}j{\text{ and serotype }}s \\ & t_{{ij}}^{\theta } :{\text{Time since vaccination at which data point }}j{\text{ for subject }}i{\text{ is observed}} \\ & \Omega ^{s} :{\text{Normalized variance - covariance matrix of the multivariate normal distribution across serotype}} \\ & \in _{{ij}} :{\text{Titre measurement error in the interval }}[ - e,e] \\ & \alpha _{g}^{s} ,\beta _{g}^{s} :{\text{Serotype and group - specific parameters of the log - logistic antibody decline function}} \\ \end{aligned}$$

Model 2 accounts for the same parameters as those included in Model 1 with the addition of an increase in antibody titers, defined as follows:

$$\left\{ {\begin{array}{*{20}l} {[Z_{{i,j}}^{s} ]\sim {\mathcal{N}}\left( {[\mu _{{i,j}}^{s} ],(t_{{i,j}} - t_{{i,j - 1}} )\Omega ^{s} } \right)} \hfill \\ {Z_{{i,j}}^{s} = X_{{i,j - 1}}^{s} - X_{{i,j}}^{s} } \hfill \\ {\mu _{{i,j}}^{s} = (1 - I_{{i,j}}^{s} )*X_{{i,j - 1}} \left( {1 - \frac{{\alpha _{g}^{s} + t_{{i,j - 1}}^{{\beta _{g}^{s} }} }}{{\alpha _{g}^{s} + t_{{i,j}}^{{\beta _{g}^{s} }} }}} \right) - I_{{i,j}}^{s} *B^{s} } \hfill \\ {X_{{i,j}}^{s} = \tilde{X}_{{i,j}}^{s} + \in _{{i,j}} } \hfill \\ { \in _{{i,j}} \sim {\text{Uniform}}( - e,e)} \hfill \\ \end{array} } \right.$$

One additional variable and one additional parameter are included in Model 2:

$$\begin{aligned} & I_{{ij}}^{s} :{\text{Observed infection for subject }}i{\text{ at time point }}j \\ & B_{i}^{s} :{\text{Serotype - specific titers post - infection for subject }}i{\text{ at data point }}j \\ \end{aligned}$$

#### Model outcomes

The posterior distributions of the model parameters allowed us to calculate for each participant the predicted evolution of log-transformed antibody titers over time (${\widehat{X}}_{i,t}^{s}$), starting from the observed post-vaccination titer. These predicted values were used to generate estimated GMTs and seroprotection rates.

$$\begin{aligned} \widehat{{GMT}}_{{g,t}}^{s} = & 2^{{\frac{1}{{n_{g} }}}} \sum\nolimits_{{i \in g}} {\tilde{X}_{{i,t}}^{s} } \\ \hat{S}_{{g,t}}^{s} = & \frac{1}{{n_{g} }}\sum\nolimits_{{i \in g}} {{\mathbf{1}}_{{[\tilde{X}_{{i,j}}^{s} > \tau ]}} } \\ \end{aligned}$$

where:

$$\begin{aligned} & \widehat{{GMT}}_{{g,t}} :{\text{Estimated serotype, group and time - specific geometric mean titer}} \\ & \hat{S}_{{g,t}} :{\text{Estimated serotype, group and time - specific seropositivity}} \\ \end{aligned}$$

$$\begin{aligned} & n_{g} :{\text{Number of subjects in study group }}g \\ & T:{\text{Threshold for seroprotection}} \\ & {\mathbf{1}}_{{[\tilde{X}_{{i,j}}^{s} > \tau ]}} :{\text{Indicator function i}}{\text{.e 1 if value }} > T{\text{ 0 otherwise}} \\ \end{aligned}$$

Estimated GMTs and seroprotection rates were compared with the observed values, to assess the quality of the fit. We also used these outcomes to predict the values at year 10.

### Statistical methods

The model was coded and analyzed in R using the RStan and loo packages^45,46^. To ensure the estimation was robust, we used a three-fold cross-validation^46,47^ stratified by group. Based on the combination of the four vaccines and the six reference studies (Table 1), a total of 11 groups were defined (Table 2). As each study is age-group specific, we used three categories for reporting results: toddlers, adolescents/young adults and older adults. Weakly informative priors were used for parameters related to antibody decline or boosting. R-hat convergence diagnostic effective sample sizes were used to assess the accuracy and robustness of model fit. Subsequently, expected log pointwise predictive densities (ELPDs)^46^, with pooled data other than the three partitions considered in the cross-validation, were used to compare the fit estimated by the different models.

#### Ethics approval

This analysis is a pooled secondary analysis based on the results of four previously published clinical studies (see Table 1). These four published clinical studies were conducted in accordance with the Declaration of Helsinki and the Quality Standards of the International Conference on Harmonization Good Clinical Practices, and were reviewed and approved by their respective ethical committees, as detailed in the respective publications.

## Results

### Study participants

The number of participants with available data at each timepoint for each age category is shown in Table 2.

### Observed persistence data

Between subsequent time points (T1 vs. T2 and T2 vs. T3), observed in each study between 3 and 7 years post-vaccination, most participants experienced a decrease in antibody titers; however, a proportion of participants experienced an antibody rise, which varied according to serogroup (A: 15.4%; C: 9.7%, W: 22.0%, Y: 11.0%, calculated by combining the data between T1 and T2 and between T2 and T3, although there were only limited data available for T3). This phenomenon is not well understood but may be explained by natural exposure to the pathogen, which is incorporated as a variable in Model 2.

**Fig. 1:**
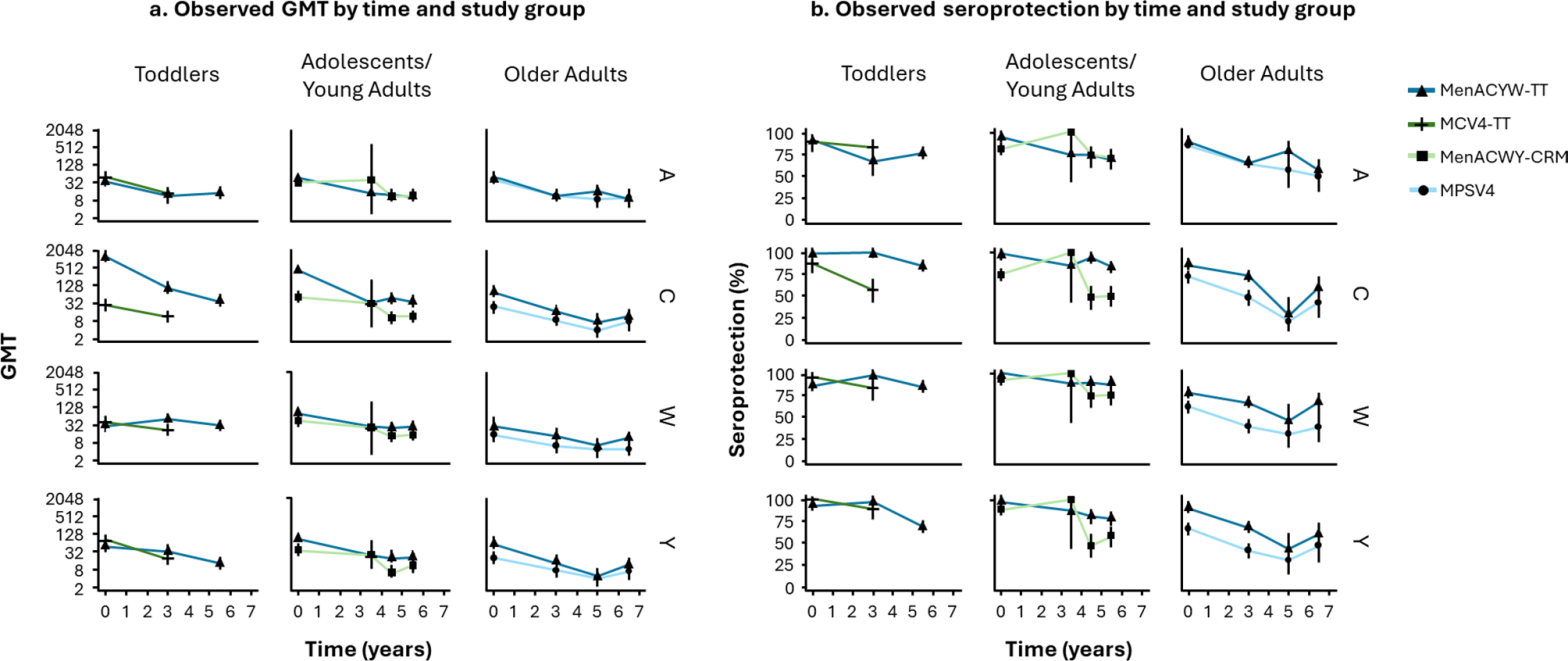
Observed (**a**) GMTs and (**b**) seroprotection rates based on hSBA ≥ 1:8, by age group and vaccine. Day 0 = 30 days after the primary vaccination. Persistence data for each age group are pooled data presented Table 1. Observations for each study and each vaccine were grouped by the number of years post-vaccination at which they have been collected (0, 3, 4 and 5 years for adolescents/young adults, 0, 3, 5 and 7 years for older adults, and 3 and 5 years for toddlers).

At the population level, GMTs and seroprotection rates for all serogroups generally decreased over time for all age groups and for each vaccine (Figure 1). At T2, GMTs were similar between vaccine groups for the respective age groups for serogroups A, W, and Y. For serogroup C, GMTs were higher at 5 and 6 years post-vaccination in adolescents/young adults and higher 3 years post-vaccination in toddlers primed with MenACYW-TT versus MenACWY-CRM or MCV4-TT, respectively. No difference for serogroup C was observed between MenACYW-TT and MPSV4 in older adults. Similarly, seroprotection rates (hSBA titer ≥1:8) were comparable between the respective vaccines across all age groups for serogroups A and W, and higher for serogroup C in adolescents/young adults and toddlers primed with MenACYW-TT versus MenACWY-CRM or MCV4-TT, respectively. No difference was observed for serogroup C between MenACYW-TT and MPSV in older adults. For serogroup Y, the seroprotection rate was higher in adolescents/young adults primed with MenACYW-TT versus MenACWY-CRM. No difference was seen for serogroup Y in older adults or toddlers primed with MenACYW-TT and MPSV4 or MCV4-TT, respectively.

### Model results

#### Observed and estimated persistence

Model 2, which specifically accounts for antibody rise in a proportion of participants, outperformed Model 1 and was therefore used as the reference model (Supplementary Table 1). The multinomial approach considered in this analyses allowed us to detect correlation in antibody decline between the four serotypes included in the vaccine. Model 2 results exhibited some notable, albeit weak, correlations for this parameter. The largest correlation (0.29) was observed between serogroups W and Y (Supplementary Table 2).

**Fig. 2:**
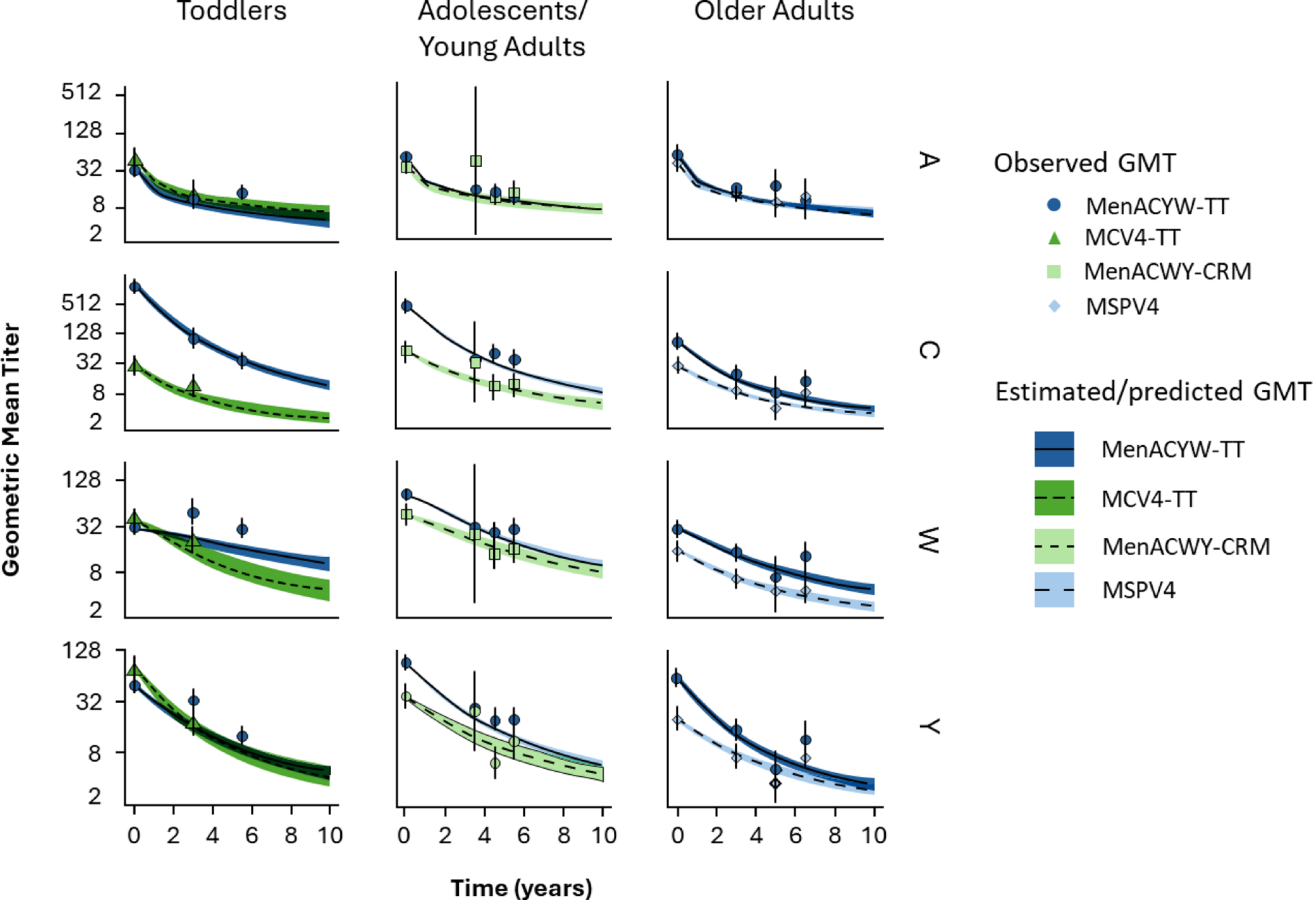
Observed and predicted GMTs according to age group and time. Day 0 = 30 days after the primary vaccination. Dots and vertical lines correspond, respectively, to observed GMT and 95% confidence interval of this observed value. Lines and corresponding shaded areas correspond, respectively, to the average estimated GMT and corresponding 95% confidence interval.

**Fig. 3:**
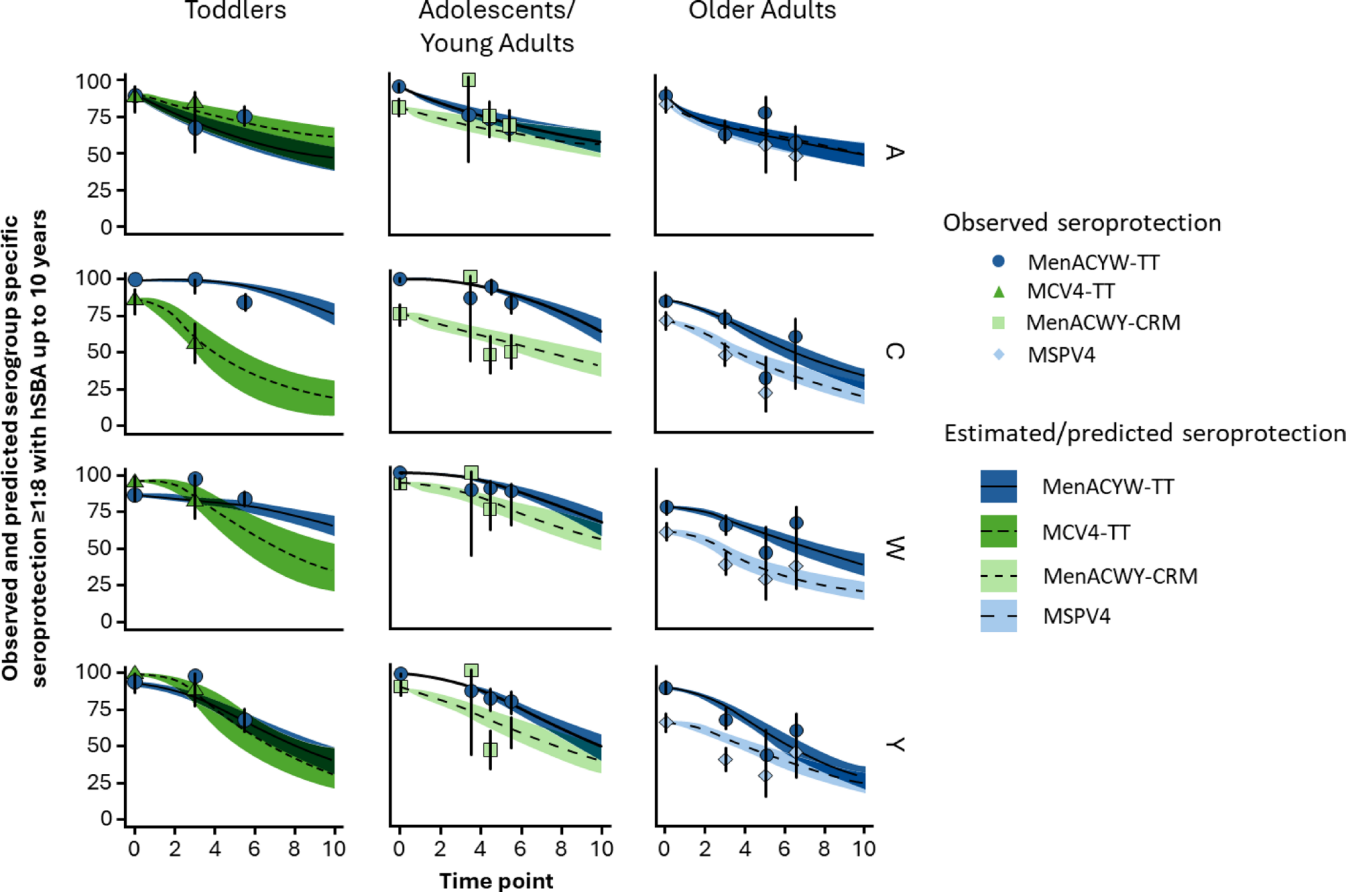
Observed and predicted serogroup-specific seroprotection rate (titer based on hSBA ≥ 1:8) up to 10 years. Day 0 = 30 days after the primary vaccination. Dots and vertical lines correspond, respectively, to observed seroprotection rate and 95% confidence interval of this observed value. Lines and corresponding shaded areas correspond, respectively, to the average estimated seroprotection rate and corresponding 95% confidence interval.

Comparison of the observed and estimated outcomes as well as the trajectory over time for the different vaccines for each serogroup are shown in Figures 2 and 3.

GMT results indicate a good fit for most data points (i.e., the estimated values obtained through cross-validation are in the range of the confidence intervals for the observed data points), although there were some outliers (serogroups W and Y for MCV4-TT). The observed antibody decline was lower for MenACYW-TT compared with MenACWY-CRM and MPSV4 in adolescents/young adults and older adults, respectively, with the exception of serogroup A, as indicated by the overlapping confidence intervals. In toddlers, there was a difference for serogroups C and W when comparing MenACYW-TT and MCV4-TT. Similar trends were observed for MenACYW-TT across all age groups.

Seroprotection outcomes exhibited similar results to those observed for GMTs, with a good fit for most data points. Differences in the antibody persistence between vaccines were mainly observed for serogroups C, W and Y.

Based on these results, we focused on predictions at year 10 to compare the antibody persistence obtained for the different vaccines considered in our analysis.

### Predicted outcomes at 10 years

#### Toddlers

**Fig. 4:**
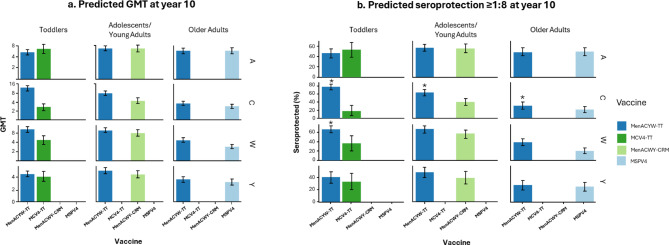
Predicted (**a**) GMTs and predicted (**b**) seroprotection rate (titer based on hSBA ≥ 1:8) at year 10. *Indicates significant differences between the predicted values for MenACYW-TT versus the comparator vaccine. Bars correspond to average predicted seroprotection rates and vertical lines to the 95% confidence interval associated with these predicted values.

Predicted seroprotection persistence rates following primary vaccination with MenACYW-TT was maintained at 10 years in 46% (95% CI 38%, 55%) of participants for serogroup A, 77% (95% CI 70%, 84%) for serogroup C, 67% (95% CI 59%, 74%) for serogroup W, and 40% (95% CI 31%, 49%) for serogroup Y (Figure 4; Supplementary Table 3). The predicted seroprotection persistence rate at 10 years was higher for MenACYW-TT compared with MCV4-TT for serogroups C (MCV4-TT 17% [95% CI 6%, 31%]) and W (MCV4-TT 36% [95% CI 20%, 53%]) (Figure 4; Supplementary Table 3). No difference was observed for the predicted seroprotection persistence rate at 10 years between MenACYW-TT and MCV4-TT for serogroups A and Y (MCV4-TT 54% [95% CI 39%, 67%] and 33% [95% CI 20%, 47%], respectively; Figure 4; Supplementary Table 3). Consistent results were observed at a seroprotection threshold of ≥1:4 (Supplementary Figure 1; Supplementary Table 4).

#### Adolescents/young adults

Predicted seroprotection persistence rates at 10 years were higher for MenACYW-TT compared with MenACWY-CRM for serogroup C (63% [95% CI 55%, 71%] vs. 40% [95% CI 32%, 48%]) and numerically higher for serogroups W (67% [95% CI 59%, 74%] vs. 57% [95% CI 47%, 67%]) and Y (49% [95% CI 40%, 57%] vs. 39% [95% CI 30%, 50%]) (Figure 4; Supplementary Table 3). The predicted antibody persistence at 10 years for serogroup A was similar in the MenACYW-TT (57% [95% CI 50%, 64%]) and the MenACWY-CRM groups (56% [95% CI 47%, 65%]). Similar results were observed at a seroprotection threshold of ≥1:4 (Supplementary Figure 1; Supplementary Table 4).

#### Older adults

Predicted seroprotection persistence rates at 10 years were higher for MenACYW-TT compared with MPSV4 for serogroups C (31% [95% CI 23%, 39%] vs. 22% [95% CI 14%, 29%]) and for serogroup W (38% [95% CI 31%, 46%] vs. 20% [95% CI 14%, 27%]) (Figure 4; Supplementary Table 3). The predicted antibody persistence at 10 years was similar between MenACYW-TT and MPSV4 for serogroups A (49% [95% CI 41%, 57%] vs. 50% [95% CI 41%, 58%]) and Y (27% [95% CI 19%, 35%] vs. 24% [95% CI 17%, 32%]). Similar results were observed at a seroprotection threshold of ≥ 1:4 (Supplementary Figure 1; Supplementary Table 4).

## Discussion

Antibody persistence data are an important aspect of the evidence reviewed for formulating recommendations on the timing of booster injections^48^ and modeling analyses are a key part in the interpretation of these data when considering evaluation of antibody decay and formulating projections to account for vaccine effectiveness^49^. This modeling analysis considered interactions between serogroups and predicted that seroprotection for each serogroup persisted at year 10 for a substantial proportion of participants vaccinated with MenACYW-TT. This analysis also showed similar, if not higher, antibody persistence for MenACYW-TT compared with MCV4-TT, MenACWY-CRM or MPSV4, respectively, particularly for serogroups C and W. When looking at specific age groups, seroprotection was higher with MenACYW-TT for serogroups C and W in toddlers and older adults compared with MCV4-TT and MPSV4, respectively, and higher with MenACYW-TT for serogroups C, W, and Y in adolescents/young adults compared with MenACWY-CRM.

The immune waning following primary vaccination reported here was expected and is consistent with the literature. Bactericidal antibody titers elicited by monovalent MenC and MCV4 vaccines have been shown to wane within 3 to 5 years after primary vaccination, with a more pronounced effect seen in younger children than in older children and adults^50–52^; however, following the initial decline, titers then stabilize between 6 and 10 years after primary vaccination^53^. Comparison of the 6–10-year persistence data between MCV4-TT and MenACWY-CRM in toddlers (aged 1 to <2 years) showed that functional antibody responses persisted 10 years after one dose of MCV4-TT, indicating long-term protection against meningococcal A, C, W, and Y disease^53^.

Compared with the exploratory analysis of Vesikari et al.^53^, the predicted seroprotection at 10 years post-vaccination (hSBA titer ≥ 1:8) reported herein for MCV4-TT in toddlers (cited as MenACWY-TT in Vesikari et al.) was higher for serogroups A, W and Y (46%, 67% and 40%, respectively vs. 26%, 44% and 41%, respectively, in Vesikari et al.). Conversely, for serogroup C, the result reported in Vesikari et al. (92%) was higher than reported here (17%). However, it should be noted that some participants in the study conducted by Vesikari et al. received an additional vaccination with a MenC vaccine if the response was deemed suboptimal, which hinders the comparison between the two studies.

Despite the anticipated waning over time^22,41,50,54,55^, the proportion of participants with an hSBA titer ≥1:8 at 10 years reported herein remained higher than the baseline values (i.e. pre-vaccination) reported in the reference articles, reflecting the situation reported in the wider published literature. Higher immunogenicity compared with baseline has been reported over 4 to 10 years following primary vaccination with MCV4-TT in toddlers (12–23 months old) and children (2–11 years old)^25,42^ at up to 5 years after primary vaccination with MenACWY-CRM in children (40–60 months old), and up to 3–5 years in infants and toddlers^50,56^. Although an antibody titer ≥1:8 is considered as a conservative serological correlate of individual protection against meningococcal disease in this analysis, any level of antibody titer may provide protection in specific circumstances^57^.

The results reported herein also include favorable predictions on the persistence of bactericidal antibody titers for older adults, an age group in which routine meningococcal vaccination is an unmet need. Aging is known to lead to a decline in immune function (immunosenescence), which may be an additional risk factor for IMD and its debilitating sequalae and consequences^58^. Although the factors underlying the acquisition and development of IMD in older adults are poorly understood, greater access to meningococcal immunization should be made a priority for clinicians and policy makers^59^.

The key strengths of this analysis were that it was robust, the fitting using cross-validation led to satisfactory and convergent results for hSBA, and there was a good match between the observed and estimated values across all data points for GMTs. Furthermore, the antibody data from the source publications were analyzed in the same laboratory, as is the case for all clinical studies investigating MenACYW-TT, and there was consistency between the seroprotection rates based on an hSBA ≥1:8 and hSBA ≥1:4. While young children and adolescents are at high risk for meningococcal disease, cases also occur at any age; for example, ten of the 90 cases notified in France in January 2025 occurred in the 5–14-year age group^60^. Therefore, given the disease severity and potential for complications, it is important to consider protection at all ages. In conjunction with other relevant evidence and, notably, the evolution of the risk of meningococcal disease according to age, the results generated here can provide useful insights for informing decisions on the timing of booster vaccinations across age ranges.

In addition, there are several limitations to this analysis that need to be acknowledged. Firstly, we conducted our pooled secondary analysis by leveraging studies that were not designed or powered to detect differences in the immune response at 10 years. As a consequence, there were a limited number of data points from each participant for use in the model and, although the estimation results were good overall, there were some outliers. Secondly, the results were based on one model, and we have not explored all potential models for antibody persistence. Finally, the results obtained with the selected reference studies might not be validated when using other datasets.

## Conclusions

In conclusion, predicted outcomes at year 10 showed similar, if not higher, immune persistence for MenACYW-TT compared with MCV4-TT, MPSV4 or MenACWY-CRM, particularly for serogroups C and W.

## Electronic supplementary material

Below is the link to the electronic supplementary material.

Supplementary Material 1.

## Data Availability

The datasets used and/or analysed during the current study available from the corresponding author on reasonable request. Further details on Sanofi’s data sharing criteria, eligible studies, and process for requesting access can be found at https://vivli.org/.
