# Supplementary material for: Modeling antibody persistence after MenACYW-TT vaccination and comparative analysis with other quadrivalent meningococcal vaccines
Source: Sci Rep. 2025 Jul 10;15:24990. doi: 10.1038/s41598-025-08112-0 (PMC12246452; doi:10.1038/s41598-025-08112-0)
Supplement: Supplementary file 1 — Supplementary Material 1. [file 41598_2025_8112_MOESM1_ESM.docx]

**Supplementary Material**

**Supplementary Table 1: Model comparison**

|  | **ELPD (difference)** | **SE (difference)** |
| --- | --- | --- |
| Model 1 | 0.0 | 0.0 |
| Model 2 | -2942.8* | 67.6 |

*The model with the best fit is defined by the highest ELPD value (ie, Model 2). ELPD, expected log pointwise predictive probabilities

**Supplementary Table 2: Correlation between serogroups in Model 2**

|  | A | C | W | Y |
| --- | --- | --- | --- | --- |
| A | 1.00 | 0.09* | 0.17* | 0.11* |
| C | 0.09* | 1.00 | 0.23* | 0.29* |
| W | 0.17* | 0.23 | 1.00 | 0.23* |
| Y | 0.11* | 0.29* | 0.23* | 1.00 |

*Significant and positive Pearson correlation (2.5%, CI >0), whereby a positive correlation between serogroups means that, at the subject level, the magnitude of the antibody decline between these serogroups are correlated.

**Supplementary Table 3: Predicted GMTs and seroprotection based on hSBA ≥1:8, by age group and vaccine at 10 years**

| **Outcome** | **Group** | **Vaccine** | **A**  **GMT (95% CI)** | **C GMT (95% CI)** | **W GMT (95% CI)** | **Y GMT (95% CI)** |
| --- | --- | --- | --- | --- | --- | --- |
| GMT | Adolescents/ young adults | MenACYW-TT | 6.68 (5.79, 7.69) | 8.01 (6.71,9.62) | 9.59 (8.10, 11.43) | 5.74 (4.90,6.75) |
| GMT | Adolescents/ young adults | MenACWY-CRM | 6.69 (5.45, 8.31) | 4.45 (3.63, 5.49) | 7.91 (6.26, 10.02) | 4.66 (3.79, 5.73) |
| GMT | Older adults | MPSV4 | 5.78 (4.84, 6.92) | 2.86 (2.43, 3.37) | 2.89 (2.48, 3.37) | 3.02 (2.59, 3.54) |
| GMT | Older adults | MenACYW-TT | 5.65 (4.79, 6.70) | 3.65 (3.11, 4.30) | 4.68 (3.97, 5.52) | 3.51 (3.00, 4.13) |
| GMT | Toddlers | MenACYW-TT | 5.27 (4.48, 6.23) | 11.89 (9.69, 14.67) | 10.27 (8.32, 12.75) | 4.72 (3.99, 5.62) |
| GMT | Toddlers | MCV4-TT | 6.43 (4.82, 8.61) | 2.67 (2.06, 3.57) | 4.65 (3.41, 6.51) | 4.11 (3.13, 5.48) |
|  |  |  | **A**  **% seroprotected (95% CI)** | **C % seroprotected (95% CI)** | **W % seroprotected (95% CI)** | **Y % seroprotected**  **(95% CI)** |
| Seroprotection | Adolescents/ young adults | MenACYW-TT | 57 (50, 64) | 63 (55, 71) | 67 (59, 74) | 49 (40, 57) |
| Seroprotection | Adolescents/ young adults | MenACWY-CRM | 56 (47, 65) | 40 (32, 48) | 57 (47, 67) | 39 (30, 50) |
| Seroprotection | Older adults | MenACYW-TT | 49 (41, 57) | 31 (23, 39) | 38 (31, 46) | 27 (19, 35) |
| Seroprotection | Older adults | MPSV4 | 50 (41, 58) | 22 (14, 29) | 20 (14, 27) | 24 (17, 32) |
| Seroprotection | Toddlers | MenACYW-TT | 46 (38, 55) | 77 (70, 84) | 67 (59, 74) | 40 (31, 49) |
| Seroprotection | Toddlers | MCV4-TT | 54 (39, 67) | 17 (6, 31) | 36 (20, 53) | 33 (20, 47) |

GMT, geometric mean titer. hBSA, serum bactericidal assay using human complement.

**Supplementary Table 4:** **Predicted GMTs and seroprotection based on hSBA ≥1:4, by age group and vaccine at 10 years**

| **Outcome** | **Group** | **Vaccine** | **A**  **GMT (95% CI)** | **C** **GMT (95% CI)** | **W** **GMT (95% CI)** | **Y** **GMT (95% CI)** |
| --- | --- | --- | --- | --- | --- | --- |
| GMT | Adolescents/ young adults | MenACYW-TT | 6.68 (6.72, 7.70) | 8.01 (6.72, 9.58) | 9.59 (8.09, 11.37) | 5.74 (4.90, 6.73) |
| GMT | Adolescents/ young adults | MenACWY-CRM | 6.69 (5.40, 8.32) | 4.45 (3.63, 5.51) | 7.91 (6.29, 10.02) | 4.66 (3.81, 5.75) |
| GMT | Older adults | MPSV4 | 5.78 (4.85, 6.91) | 2.86 (2.45, 3.37) | 2.89 (2.50, 3.37) | 3.02 (2.58, 3.54) |
| GMT | Older adults | MenACYW-TT | 5.65 (4.80, 6.70) | 3.65 (3.12, 4.31) | 4.68 (3.98, 5.53) | 3.51 (3.01, 4.12) |
| GMT | Toddlers | MenACYW-TT | 5.27 (4.49, 6.22) | 11.89 (9.70, 14.56) | 10.27 (8.33, 12.79) | 4.72 (3.99, 5.62) |
| GMT | Toddlers | MCV4-TT | 6.43 (4.85, 8.68) | 2.67 (2.06, 3.53) | 4.65 (3.42, 6.48) | 4.11 (3.13, 5.44) |
|  |  |  | **A**  **% seroprotected (95% CI)** | **C** **% seroprotected (95% CI)** | **W** **% seroprotected (95% CI)** | **Y** **% seroprotected (95% CI)** |
| Seroprotection | Adolescents/ young adults | MenACYW-TT | 84 (79, 88) | 87 (82, 94) | 91 (86, 94) | 79 (73, 84) |
| Seroprotection | Adolescents/ young adults | MenACWY-CRM | 79 (73, 85) | 63 (56, 70) | 82 (75, 89) | 68 (59, 76) |
| Seroprotection | Older adults | MenACYW-TT | 76 (70, 82) | 48 (40, 56) | 46 (38, 54) | 50 (43, 58) |
| Seroprotection | Older adults | MPSV4 | 76 (71, 82) | 60 (52, 67) | 67 (59, 73) | 59 (50, 67) |
| Seroprotection | Toddlers | MenACYW-TT | 77 (70, 84) | 93 (90, 96) | 86 (82, 90) | 73 (65, 79) |
| Seroprotection | Toddlers | MCV4-TT | 80 (69, 90) | 45 (31, 61) | 70 (53, 84) | 63 (49, 78) |

GMT, geometric mean titer. hBSA, serum bactericidal assay using human complement.

**Supplementary Figure 1: Serogroup-specific observed and predicted seroprotection based on hSBA ≥1:4**


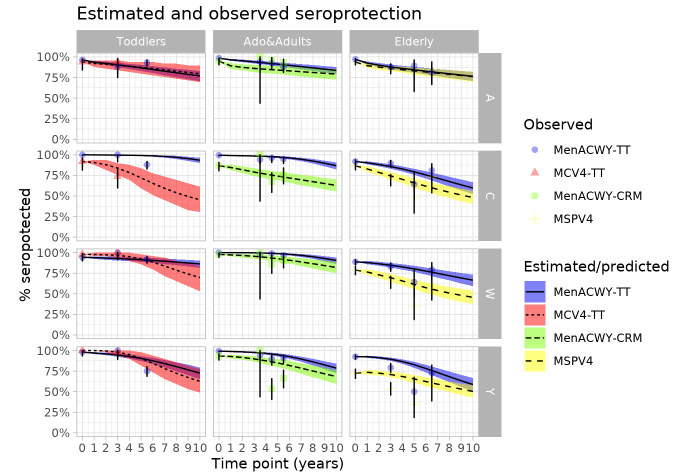


Day 0 = 30 days after the primary vaccination. Dots and vertical lines correspond, respectively, to observed seroprotection rate and 95% confidence interval of this observed value. Lines and corresponding shaded areas correspond respectively to the average estimated seroprotection rate and corresponding 95% confidence interval.

hBSA, serum bactericidal assay using human complement
